# Current status of diagnosis and Mesenchymal stem cells therapy for acute pancreatitis

**DOI:** 10.14814/phy2.14170

**Published:** 2019-11-06

**Authors:** Fahad Munir, Muhammad B. Jamshed, Numan Shahid, Syed A. Muhammad, Noor B. Ghanem, Zhang Qiyu

**Affiliations:** ^1^ Department of Hepatobiliary Surgery The First Affiliated Hospital of Wenzhou Medical University Wenzhou People’s Republic of China; ^2^ Department of General Surgery The School of International Studies of Wenzhou Medical University Wenzhou People’s Republic of China; ^3^ Institute of Molecular Biology and Biotechnology Bahaudin Zakariya University Multan, Punjab Pakistan; ^4^ The School of International Studies of Wenzhou Medical University Wenzhou People’s Republic of China

**Keywords:** Acute pancreatitis, inflammation, mesenchymal stem cells, systemic inflammatory response syndrome

## Abstract

Acute pancreatitis (AP) is an acute gastrointestinal disorder that is the most common and requiring emergency hospitalization. Its incidence is increasing worldwide, thus increasing the burden of medical services. Approximately 20% of the patients develop moderate to severe necrotizing pancreatitis associated with pancreatic or peri‐pancreatic tissue necrosis and multiple organ failure. There are many reports about the anti‐inflammatory effect of mesenchymal stem cells (MSCs) on pancreatitis and the repair of tissue damage. MSCs cells come from a wide range of sources, autologous MSCs come from bone marrow and allogeneic MSCs such as umbilical cord blood MSCs, placenta‐derived MSCs, etc. The wide source is not only an advantage of MSCs but also a disadvantage of MSCs. Because of different cell sources and different methods of collection and preparation, it is impossible to establish a unified standard method for evaluation of efficacy. The biggest advantage of iMSCs is that it can be prepared by a standardized process, and can be prepared on a large scale, which makes it easier to commercialize. This paper reviews the present status of diagnosis and progress of MSCs therapy for AP.

## Introduction

Acute pancreatitis (AP) is an aggressive disorder of exocrine pancreatic cells. AP is an acute gastrointestinal disorder that is the most common and requiring emergency hospitalization. Its incidence is increasing worldwide, thus increasing the burden of medical services (Lankisch, Apte and Banks, 2015). In the past decades, the management of AP has progressively developed into an individualized multi‐disciplinary approach. Endoscopy, imaging examination, and surgical intervention play an essential role in the treatment strategy. However, effective and specific treatment for SAP is still unavailable. At present, MSCs have been used in many animal models and human clinical trials to study the effects of MSCs transplantation for digestive tract diseases, including inflammatory bowel disease (Pérez‐Merino et al., 2015), cirrhosis (Volarevic et al., 2014), ischemia‐reperfusion injury (Wise et al., 2014). The immunomodulatory effects of MSCs on the secretion of various anti‐inflammatory molecules (Hynes et al., 2016). MSCs have great potential in cell therapy not only with low immunogenicity and immune regulation but also with multidirectional differentiation, directional migration, tissue repair, and inhibition of inflammation damage (Deak, Seifried and Henschler, 2010). This article reviews the current status of diagnosis and progress of MSCs therapy for AP.

## Diagnosis of early AP

Based on RAC criteria in 2012, more than two of the following criteria for the diagnosis of AP should meet (1) Abdominal pain: most of the patients are acute onset, 80–85% of the patients may have abdominal pain symptoms, mainly pain in the upper abdomen, often radiate to the back. (2) The elevation of serum amylase or lipase: is usually three times higher than the upper cut‐off value. (3) The symptoms of AP shown by CT and it also relies on MRI or color Doppler ultrasound to assist diagnosis: For typical clinical manifestations, laboratory diagnostic evidence is required to confirm the diagnosis and no additional enhanced CT or MRI is needed. Mild, moderate or severe are the classification of AP. When there are no local complications, they are mild or moderate (e.g., peri‐pancreatic exudation), systemic complications (e.g., deterioration of chronic diseases), temporary (<48 h) organ failure or persistent (>48 h) organ failure AP is severe in nature (Thoeni, 2012).

## Etiology

**Figure 1:**
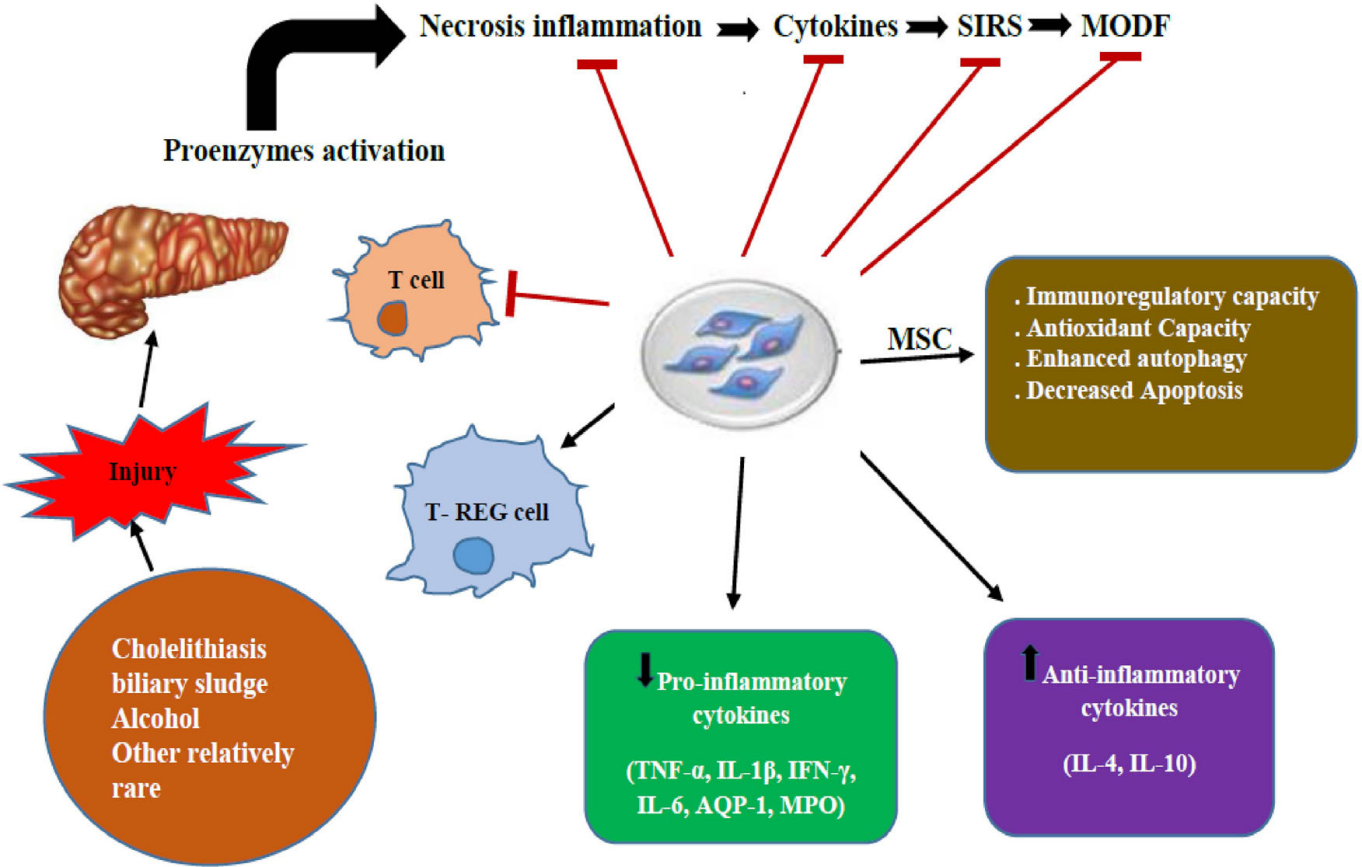
Illustrate the overall relationships of mesenchymal stem cells (MSCs) therapy for acute pancreatitis (AP).

Cholelithiasis and biliary sludge are the main causes of AP and it accounts for about 40–50% of cases in western countries. (Yadav and Lowenfels, 2006; Yadav and Lowenfels, 2013) About 20% of cases are caused by alcohol, which is the second leading cause of AP (Apte, Pirola and Wilson, 2010; Coté et al., 2011; Whitcomb et al., 2012). Other relatively rare causes include drug therapy (Nitsche et al., 2012), ERCP (Cotton et al., 2014), hypercalcemia (Bai et al., 2012), hypertriglyceridemia (Stefanutti, Labbadia and Morozzi, 2013), surgery and trauma (Fitzpatrick, 2017) (Fig. 1). It is essential to define the cause of AP in a standard diagnostic and therapeutic process, as it partially promotes early disease management and follow‐up treatment strategies. Standard treatment procedures include inquiry about medical history, physical examination, laboratory examination (amylase, triglyceride, calcium) and USG (Working, Group IAP, and Acute Pancreatitis Guidelines APA, 2013). AP with unknown etiology accounts around about 10–25% of cases. The diagnosis of idiopathic pancreatitis requires multiple abdominal USG and EUS (Somani, Sunkara and Sharma, 2017). Meta‐analysis showed that about 61% of the cases could be diagnosed by EUS. These contain a finding of microorganisms, gallstones or biliary sludge (41%), and chronic pancreatitis or pancreatic tumors are the other causes of AP (Somani, Sunkara and Sharma, 2017).

## Estimation of severity

Identification of patients at low or high risk of complications by severity prediction of AP. This is valuable for proper monitoring of graded patients and for stratifying patients into RCTs. Several scoring systems determine the serious process of disease by evaluation of clinical and laboratory results: APACHE‐II (Bezmarević et al., 2012), Ranson score (Aphinives et al., 2011; Tan et al., 2017), improved Glasgow‐Imrie criteria (Tan et al., 2017), SIRS standard, bedside index of AP severity and noninvasive AP score (Gao, Yang and Ma, 2015), whereas CRP might also be used as an indicator of disease severity (Zheng, Zhang and Gao, 2015). About 50% of patients predict mild AP. While 50% of them will eventually develop moderate or SAP. The mortality rate of SAP is predicted to be about 10%, while that of mild pancreatitis is predicted to be <1%. Therefore, the scoring system is mainly used to exclude the possibility of progressing SAP. Because the numerous predictive scoring systems represent a similar score, the IAP/APA guidelines recommend the use of continuous SIRS (>48 h) as a predictor of SAP because of its simplicity (Uhl et al., 2002; Working et al., 2013).

## Management of acute phase

Since there is no treatment for AP, early treatment includes supportive treatment, including pain management and adequate fluid resuscitation (Working et al., 2013). Pancreatic and following systemic inflammation leads to fluid exudation into the third space. This may lead to low blood volume, low perfusion, and eventual organ failure in severe cases. Sufficient fluid resuscitation is needed to counteract this cascade of inflammation (Zheng, Zhang and Gao, 2015). Limited RCTs have investigated the types of liquids. Colloidal rehydration is not encouraged in general critically ill patients because there is no evidence to support its effectiveness and even mortality rate increases with hydroxyethyl starch. Therefore, the IAP/APA guidelines recommend liquid resuscitation using crystalline solutions such as Ringer's lactate solution (Working et al., 2013). This is a multicenter RCTs based on 40 patients with AP. The results showed that compared with saline, Ringer's lactate solution improved CRP level and SIRS (Zheng, Zhang and Gao, 2015). Ringer's lactate or Plasma‐Lyte solution advantage has not been studied by larger RCTs in patients with saline‐induced pancreatitis. Further research is needed, as many RCTs in ICU have failed to find significant results when using equilibrium fluids.

## Fluid resuscitation

Five RCTs have been conducted with different fluid resuscitation schemes. Two RCTs in 76 and 115 patients with SAP showed quick, uncontrolled fluid resuscitation (10–15 mL/kg/h) or hematocrit <35% within 48 h considerably increased the incidence of infection, abdominal syndrome, mechanical ventilation requirement, and subsequently increased the death rate (Mole et al., 2011).

In a RCT of mild pancreatitis, 60 patients were predicted Ringer's lactate solution (20 mL/kg intravenous injection 3 mL/kg/h micro‐pump) and Ringer's lactate solution standard hydrate (10 mL/kg intravenous injection 1.5 mL/kg/h micro‐pump) improved the composite endpoint (i.e., "medical improvement within 36 h") (Mao et al., 2009). Therefore, too much or too little fluid infusion during fluid resuscitation is harmful, largely depending on the severity of pancreatitis. For instance recommended in the IAP/APA guidelines, appropriate monitoring and early goal‐directed fluid therapy may still be recommended treatment (; Working et al., 2013). Early target‐directed fluid therapy is a multicenter RCT of 40 patients with AP (Mao et al., 2009) and a RCT of 200 patients with SAP (Mole et al., 2011). The first RCT was unable to confirm the advantage of early‐decided fluid therapy; on the other hand, in the whole RCT group, the incidence of SIRS was very low, indicating that there were few SAP patients.

The second RCT suggested that in the early target‐directed fluid therapy group mechanical ventilation time, morbidity, and mortality of multiple organ failure were reduced, especially when FFP was administrated during fluid resuscitation. Nonetheless, the baseline of APACHE‐II score in the control group was significantly worse, suggesting that randomization was not balanced enough, and further RCTs were needed. From these RCTs, it is difficult to conclude because the parameters (fluid type, fluid protocol, and recovery target) are different from each other. Since hydration state by a single parameter is not fully revealed thus observation of the multiple parameters are suggested. In hospitalization, the key monitoring indicators included heart rate <120/min, mean arterial pressure between 64–85 mmHg, and urine volume at least 0.5 mL/kg/h. Significant laboratory outcomes, including creatinine levels, BUN, and hematocrit, must be maintained between 35 and 44% (Van DIjk et al., 2017).

## Pain management

The main symptom of AP is pain and should be cured promptly and effectively. It is indicated that pain scores are often reassessed, the type and/or dose of analgesics need to be adjusted to ensure suitable pain management. Numerous RCTs compared the effects of different kinds of analgesia in AP (Blamey et al., 1984; Jakobs et al., 2000; Stevens, Esler and Asher, 2002; Kahl et al., 2004; Peiró et al., 2008; Wilms, Meffert and Schultes, 2010; Layer et al., 2011). A recent meta‐analysis report indicated that opioid use in AP and most RCTs are of low quality and do not have specific analgesic strategies (Basurto Ona, Rigau Comas and Urrútia, 2013). Because the recent evidence is inadequate, pain can be treated with the most advanced pain protocol.

## Preventive use of antibiotics and probiotics

Secondary infection of the pancreas or peri‐pancreatic necrosis is one of the utmost lethal complications of AP, which is considered the outcome of intestinal bacterial translocation. (Bradley, 1993) Some double‐blind RCTs did not show prophylactic use of antibiotics in the treatment of pancreatic necrotizing infections (Isenmann et al., 2004; Dellinger et al., 2007; García‐Barrasa et al., 2009), as confirmed by meta‐analysis (Villatoro, Mulla and Larvin, 2010; Lim et al., 2015). Therefore, antibiotics are only used when infection is confirmed or clinically suspected. Many studies have attempted to use probiotics to influence intestinal microflora, to prevent bacterial translocation. Two RCTs compared probiotics and placebo in 62 and 45 patients with SAP and reported encouraging outcomes (Oláh et al., 2002; Oláh et al., 2007). However, a subsequent multicenter RCTs of 296 patients with SAP treated with probiotics predicted increased mortality and incidence of non‐occlusive mesenteric ischemia (Besselink et al., 2008). Hence, the treatment with probiotics at present is considered as a contraindication for the treatment of SAP.

## Nutritional support

Enteral nutrition in patients is not only provided enough intake of calories but also improves clinical results. It is speculated that the combination of poor intestinal peristalsis, increased intestinal mucosal permeability, overgrowth of bacteria and intestinal bacterial translocation, which may develop a secondary infection of the pancreas (Van Felius et al., 2003). Enteral nutrition can decrease translocation and bacterial overgrowth by stimulating intestinal peristalsis, thus maintaining intestinal mucosal integrity (Nieuwenhuijs et al., 1998; Rahman et al., 2003; Fritz et al., 2010). Cochrane's evaluation of eight RCTs confirmed this. Compared with conventional total parenteral nutrition, 348 patients receiving conventional enteral nutrition for AP had a lower infection rate, organ failure, and death rate (Besselink et al., 2009). In addition, the timing of enteral nutrition may be very important. Many retrospective studies suggest that early nasal feeding can considerably reduce the infection rate (Guillou, 1999; McClave and Heyland, 2009; Marik, 2009). A multicenter RCTs of 208 patients with SAP was conducted to compare the effects of early nasogastric jejunal feeding (<24 h) and oral feeding after 72 h (nasogastric jejunal feeding as required) on infection or death rate. Notably, 69% of patients did not receive a nasal feeding tube in the control group, to avoid the possible discomfort (Bakker et al., 2014). Another RCTs compared 214 patients who received early nasogastric jejunal feeding (<24 h) without nutritional support and showed the benefits of early nutritional support (Wereszczynska‐Siemiatkowska et al., 2013). According to these RCTs, it is anticipated that tube feeding for SAP will be restricted to patients with inadequate oral calorie intake after 3–5 days.

It was described that nasal feeding in AP increased the risk of aspiration, inflammation, and pain due to stimulation of pancreatic secretion. A number of RCTs stated that nasojejunal feeding was not superior to nasal feeding (Eatock et al., 2005; Kumar et al., 2006; Bakker et al., 2014; Stimac et al., 2016). Therefore, now it is considered that both enteral nutrition methods are safe and feasible (Singh et al., 2012; Chang et al., 2013). In patients with mild pancreatitis, many RCTs showed that once the pain was reduced, oral diet could be restored (Eckerwall et al., 2007).

## Application of ERCP in biliary pancreatitis

Temporary obstruction at the Ampulla of Vaters with acute biliary pancreatitis is considered to cause pancreatic inflammation. If the biliary obstruction is not relieved, it may aggravate the course of the disease. Early biliary decompression, ERCP, and ES have been comprehensively explored as potential interventions to enhance medical results in biliary pancreatitis (Fölsch et al., 1997; Oría et al., 2007; Moretti et al., 2008).

## Timing of drainage for infectious necrotizing pancreatitis

For (suspected) infectious necrotizing pancreatitis in patients with the standard invasive management is also a so‐called "ladder" approach (Van Baal et al., 2011; Van Santvoort et al., 2011; Da Costa et al., 2014). This procedure could be performed by surgery (including percutaneous drainage) or endoscopy (involving endoscopy, usually via the stomach, drainage) (Zhu, Fan and Zhang, 2001; Horvath et al., 2001; Aranda‐Narváez et al., 2014). Current guidelines suggest that invasive interventions must be delayed until infection (additional) with pancreatic necrosis has developed a "wrapping" process, generally 3–4 weeks after onset (Beger et al., 1988; Traverso and Kozarek, 2005; Seifert et al., 2009). The debridement of non‐capsular necrosis is technically challenging and carries a high risk of hemorrhage and perforation to adjacent organs. In these 3 to 4 weeks, patients are usually very ill and need to be admitted to ICU; however, in most of these patients catheter drainage is technically feasible. There is a question of worth discussing when catheter drainage would be carried out before the stage of encapsulated necrosis, especially since without any additional necrotic tissue removal about 30% of patients can recover after catheter drainage (Rodriguez et al., 2008). Catheter drainage as the preferred step‐by‐step method may delay catheter drainage until a package is formed in the necrotic area, which in fact, may slow down the recovery process. Early detection of necrotic areas of infection and catheter drainage may improve prognosis, but there is still a lack of strong data support.

## Stem cell therapy for AP

In the past decades, the management of AP has progressively developed into an individualized multi‐disciplinary approach. Endoscopy, imaging examination, and surgical intervention play an essential role in the treatment strategy. However, effective and specific treatment for SAP and CP is still unavailable. The mortality rate of SAP is still high, which seriously threatens the life safety of patients. All of these urgently need us to find a new direction or research ideas. At present, MSCs have been used in many animal models and human clinical trials to study the effects of MSCs transplantation for digestive tract diseases, including inflammatory bowel disease (Pérez‐Merino et al., 2015), cirrhosis (Volarevic et al., 2014), ischemia‐reperfusion injury (Wise et al., 2014). The immunomodulatory effects of MSCs on the secretion of various anti‐inflammatory molecules. MSCs do not have MHC class II antigen thus do not reject even after allotransplantation (Hynes et al., 2016). MSCs have great potential in cell therapy not only with low immunogenicity and immune regulation but also with multidirectional differentiation, directional migration, tissue repair and inhibition of inflammation damage (Deak, Seifried and Henschler, 2010). The anti‐inflammatory properties of MSCs are considered to be an effective treatment for AP and CP. MSCs can not only inhibit the occurrence of inflammatory reaction and alleviate the injury of the pancreas itself, but also alleviate the injury of organs other than pancreas in severe pancreatitis, so as to achieve the goal of treating acute pancreatitis (Fig. 1).

## MSCs alleviate pancreatic injury by inhibiting the inflammatory response

MSCs come from a wide range of sources, including umbilical cord, bone marrow, fetal membranes, and adipose tissue. Allogeneic and autologous MSCs transplantation have been accomplished. Jung et al. first reported MSCs therapy for AP in 2009 (Jung et al., 2011). They assessed the effects of MSCs transplantation from human bone marrow on mild and SAP in rats. MSCs can reduce the expression of inflammatory markers such as TNF‐a, IL‐1beta, and IL‐6 in pancreas on the one hand, and upsurge the Fox‐p3 positive regulatory T cells in lymph nodes and pancreas on the other hand (Tu et al., 2012). Subsequent studies have also confirmed that MSCs have anti‐inflammatory properties by inhibiting the levels of pro‐inflammatory cytokines in serum and pancreatic tissue, increasing the expression of anti‐inflammatory makers, and regulating the balance between pro‐inflammatory and anti‐inflammatory factors (Yang et al., 2013). MSCs transplanted immediately after AP induction had a better anti‐inflammatory effect than MSCs transplanted numerous hours after AP induction. It was confirmed that the anti‐inflammatory properties of cell therapy of MSCs were positively correlated with the time and dose of action (Fig. 1). Another mechanism of MSCs inhibiting AP inflammation is the anti‐apoptotic effect (Meng et al., 2013). By blocking JNK pathway, MSCs regulate the transcription of downstream apoptosis‐related target genes and the expression of apoptotic proteins in a transcription‐dependent manner. MSCs mediate apoptosis of acinar cells through death receptor protrusion and mitochondrial pathway. In addition, MSCs can promote the repair of damaged tissues and angiogenesis through SDF‐1a/C‐X‐C chemokine receptor type 4 (CXCR4) axis (Qian et al., 2015) A few bioactive molecules secreted by MSCs play a significant role in the regulation of inflammatory immunity although the exact mechanism of anti‐inflammatory effects of MSCs is still controversial. It was previously reported that microcapsules from MSCs could alleviate AP‐induced injury (Sala et al., 2015; Yin et al., 2016).

After sodium deoxycholate treatment, the pancreatic acinar cells survival rate was decreased significantly, the levels of amylase and lactate dehydrogenase in cell supernatant increased significantly, while the activity of SOD decreased significantly, and the level of MDA increased significantly. When MSCs were co‐cultured, the pancreatic acinar cells survival rate was increased significantly, the secretion rate of amylase and the leakage rate of lactate dehydrogenase decreased significantly, and the oxidative stress response was significantly alleviated (Tu et al., 2012). In addition, SD rat models of mild and SAP were induced by different experimental methods, and then MSCs were injected into tail vein of rats to observe the therapeutic effect of MSCs on different degrees of acute pancreatitis (Jung et al., 2011). The results showed that MSCs could migrate to damaged pancreatic tissue specifically. Confocal results also suggested that the migration of MSCs might be related to the degree of inflammation. In the SAP group, after MSCs intervention, the levels of biochemical indicators such as amylase and lipase in pancreatitis also decreased significantly. Pathological results also indicated that the hyperemia and edema of pancreatic tissue, the infiltration of inflammatory cells, parenchymal hemorrhage, necrosis and apoptosis of acinar cells were significantly alleviated. Similarly, in mild pancreatitis group, namely edematous pancreatitis group, MSCs also effectively alleviated the degree of pancreatic edema. In addition, MSCs significantly inhibited the activity of MPO and effectively reduced the oxidative stress effect caused by peroxidase (Jung et al., 2011). More studies have found that MSCs intervention at the onset of SAP can more effectively alleviate pancreatic injury, reduce SIRS level, decrease the incidence of external organ damage, and low the mortality rate (Yang et al., 2013), compared with MSCs intervention before and several hours after the onset of SAP (Fig. 1). In addition, compared with 5 × 104 cells/kg, 5 × 105 cells/kg, 5 × 106 cells/kg and 1 × 107 cells/kg groups, 5 × 106 cells/kg group can play a better therapeutic role. The results showed that MSCs had a certain time and dose dependence on the treatment of SAP (Yang et al., 2013).

## MSCs can effectively alleviate extra‐pancreatic organ damage

Lung and kidney are the most frequently damaged extra‐pancreatic organs in SAP. In the early stage of AP, TNF‐a and substance P play a significant role in the occurrence of acute lung injury caused by SAP. On the one hand, elastase activation can induce TNF‐a‐mediated acute lung injury, while in the same experimental environment; the incidence of acute lung injury is significantly reduced (Jaffray et al., 2000). On the other hand, TNF‐a can stimulate the release of MMP 9 from polynuclear leukocytes, which further induces the migration of polynuclear leukocytes and leaks out of the alveolar‐capillary barrier (Qi et al., 2014). Substance P is a gene product of protachykinin A, a neuropeptide that regulates different stages of inflammation. In the course of lung injury caused by AP, pulmonary epithelial cells and alveolar epithelial cells are injured, resulting in alveolar vacuolar membrane damage and microvascular fluid leakage, and then pulmonary interstitial edema occurred (Akbarshahi et al., 2012). Therefore, in regulating the severity of AP and pancreatitis‐related lung injury substance P plays a significant role. As a result, of these inflammatory factors, the permeability of pulmonary vascular epithelial barrier increases excessively, accompanied by fluid infiltrations into alveolar space and interstitial lung, progressing to pulmonary edema and dyspnea, by accompanied with ARDS (Zhou et al., 2010). MSCs do not only effectively alleviate alveolar, interstitial edema, reduce bleeding, reduce inflammatory cells infiltration and alleviate the damage of pulmonary lobe structure, but also effectively reduce the level of TNF‐a and substance P, and repair damaged lung tissue by inhibiting inflammatory response (Wang et al., 2012). The over‐stimulation and expression of inflammatory markers and the changes of vascular intimal barrier permeability play a key role in the incidence and development of SAP. A great number of inflammatory factors activate the blood vessel endothelial permeability, and even the barrier is broken. The macromolecule material in the blood vessel seeps out from the blood vessel and flows into the tissue gap. Nowadays, there are systemic edema and relatively insufficient blood volume, which leads to insufficient blood perfusion of important organs, such as heart, lung, kidney, brain and so on. As the disease progresses, these hemodynamic changes may lead to hemorrhagic dysfunction or even failure of important organs. Therefore, in addition to inhibiting the over‐activation and expression of inflammatory mediators, that is, blocking the SIRS process in the early stage, actively and effectively improving microcirculation is also an effective way to improve the course of SAP, that is, early fluid target resuscitation, and also an important means to reduce mortality (Foitzik et al., 2000).

In the rat model, ANP can induce significant downregulation of AQP in pancreas, lung and small intestine vascular endothelial cells, which plays an essential role in mediating water transport (Engel, Fujiyoshi and Agre, 2000; Verkman, 2002; Feng et al., 2012). AQP‐1 is mainly expressed in proximal renal tubular epithelial cells. Abnormal expression of AQP‐1 may cause water reabsorption and filtration dysfunction (Verkman, 2002). MSCs intervention can significantly alleviate the inhibition of AQP‐1 expression caused by SAP (Fig. 1), thereby alleviating water reabsorption and filtration disorders (Chen et al., 2013). MSCs added into the tail vein of established SAP rat model showed that MSCs not only significantly decreased serum amylase, urea nitrogen, and creatinine levels but also significantly improved the permeability of renal vascular endothelial cells by transmission electron microscopy, thereby effectively alleviating the injury of renal vascular endothelial cells (Chen et al., 2013). AP small intestine damage is mainly due to microcirculation disorders, secondary blood loss to tissue gap, resulting in insufficient effective blood volume, visceral vasoconstriction, resulting in the small intestine and other organs of ischemia‐reperfusion injury. After intervention with MSCs, it was found that MDA and SOD levels in serum and tissues were decreased, inflammatory factors such as TNF‐a, IL‐6, and IL‐1beta 1 in serum were down‐regulated, AQP‐1 expression in intestinal epithelial cells was increased, intestinal permeability was decreased, intestinal epithelial cell recovery was promoted, intestinal barrier integrity was maintained, intestinal injury in SAP was improved (Lu et al., 2017). In addition, MSCs can effectively alleviate the rupture of lamina propria (capillary exposure and local bleeding), inhibit inflammatory cell infiltration, and increase the survival rate of villous cells and lamina propria glands in the small intestine (Tu et al., 2012). Recalling a series of clinical trials and basic research on the treatment of pancreatitis, we can find that MSCs have great potential in the management of AP and SAP. As reported by the research institute, it is also a good breakthrough in the application of inflammation. SIRS response is an unavoidable pathophysiological process in the progress of SAP. Finding a way to negatively regulate SIRS will eventually be a breakthrough to reduce the mortality of SAP. If it can be achieved, it will eventually become the next milestone of SAP treatment.

## Summary

MSCs can play a significant role in the cure of AP and SAP by its own immune regulation.

## Conflict of Interest

None declared.
